# Efficacy and Safety of Avatrombopag in Patients With Thrombocytopenia: A Systematic Review and Meta-Analysis of Randomized Controlled Trials

**DOI:** 10.3389/fphar.2019.00829

**Published:** 2019-07-26

**Authors:** Chunlu Li, Xiaoxuan Li, Feihong Huang, Jing Yang, Anguo Wu, Long Wang, Dalian Qin, Wenjun Zou, Jianming Wu

**Affiliations:** ^1^Department of Chinese Materia Medica, School of Pharmacy, Chengdu University of Traditional Chinese Medicine, Chengdu, China; ^2^Laboratory of Chinese Materia Medica, Department of Pharmacology, School of Pharmacy, Southwest Medical University, Luzhou, China; ^3^Department of Pharmacy, The Second People’s Hospital of Yibin, Yibin, China; ^4^Institute of Cardiovascular Research, The Key Laboratory of Medical Electrophysiology, Ministry of Education of China, Collaborative Innovation Center for Prevention and Treatment of Cardiovascular Disease of Sichuan Province, Medical Key Laboratory for Drug Discovery and Druggability Evaluation of Sichuan Province, Luzhou Key Laboratory of Activity Screening and Druggability Evaluation for Chinese Materia Medica, Luzhou, China; ^5^Department of Pharmacy, Affiliated Hospital of Southwest Medical University, Luzhou, China

**Keywords:** avatrombopag, thrombocytopenia, TPO, thrombopoietin receptor agonist (TPO-RA), platelet, systematic review

## Abstract

**Background:** Avatrombopag is a novel oral, nonpeptide thrombopoietin receptor agonist (TPO-RA). A few studies have shown that avatrombopag is effective against thrombocytopenia. However, no systematic review has been conducted on the efficacy and safety of avatrombopag. Therefore, the aim of this study was to comprehensively assess the efficacy and safety of avatrombopag patients with thrombocytopenia.

**Methods:** Databases including Medline, PubMed, Embase, the Cochrane Library and ClinicalTrials.gov were searched for randomized controlled trials that compared avatrombopag with placebo in patients with thrombocytopenia. The deadline was March 2019.

**Results:** In total, 743 patients were analyzed in five clinical trials. Patients treated with avatrombopag achieved higher platelet response (OR: 17.71, 95% CI [11.01 to 28.48], *p* < 0.00001) than with placebo. Avatrombopag produced an absolute increment in platelet count (WMD: 31.13%, 95% CI [22.27 to 39.99], *p* < 0.00001) unlike the placebo. In addition, the incidence of serious adverse events (RR: 1.18, 95% CI [0.72 to 1.93], *p* = 0.51) and deaths (RR: 0.93, 95% CI [0.19 to 4.45], *p* = 0.93) in patients treated with avatrombopag was not significantly different from that in patients treated with placebo. The incidence of adverse events in patients treated with avatrombopag was slightly higher than that in patients treated with placebo (RR: 1.25, 95% CI [1.05 to 1.49], *p* = 0. 01) after one trial with high heterogeneity was removed.

**Conclusions:** This meta-analysis showed that avatrombopag was an effective treatment for thrombocytopenia, but there is sufficient evidence to indicate that adverse events may occur.

## Introduction

Thrombocytopenia is defined as a platelet below 150 × 10^9^/L, a platelet count between 70 × 10^9^/L and 150 × 10^9^/L is considered mild thrombocytopenia, and a count below 50 × 10^9^/L is considered severe thrombocytopenia. Most individuals are asymptomatic if their platelet count is 50 × 10^9^/L or higher (Gauer and Braun, 2012). Patients with a platelet count below 50 × 10^9^/L are more prone to spontaneous bleeding (i.e., mucosal, gastrointestinal, genitourinary, and intracranial bleeding), which is considered as a hematologic emergency (Stasi, 2012; Gado and Domjan, 2014). There are several causes of thrombocytopenia, including infection, malignancy, autoimmune disease, liver disease, disseminated intravascular coagulation, drugs, pregnancy, and coagulopathy (Smock and Perkins, 2014). Among these, severe thrombocytopenia may be associated with severe mortality, cancer, immune thrombocytopenic purpura (ITP) (Lambert and Gernsheimer, 2017), chronic liver disease (CLD) (Giannini et al., 2003), chronic hepatitis C virus (HCV) (Dienstag and McHutchison, 2006), and other diseases. Fundamental ITP is an acquired immune-mediated disease was characterized by autoantibody-mediated platelet destruction and impaired platelet production. Generally, these dual effects lead to severe thrombocytopenia and bleeding tendency, with associated morbidity and mortality (Bussel et al., 2014; Taylor et al., 2017). Between 2 and 4 out of 100,000 adults develop ITP, which results in bleeding symptoms and thrombocytopenia. Female adolescents are more susceptible to ITP than males (Lambert and Gernsheimer, 2017). A study conducted in Denmark reported that patients with ITP had a 1.5-fold higher mortality and a significantly increased risk of bleeding, hematologic malignancy, and infection (RR: 2.4, 5.7, and 6.2, respectively). In addition, demographic studies have shown higher mortality in patients with ITP than in the general population and that mortality risk may be associated with disease severity (Frederiksen et al., 2014). Thrombocytopenia is a common complication of patients with CLD, and occurs in up to 76% of patients with cirrhosis (Lu et al., 2006). Traditional therapies for thrombocytopenia seek to reduce platelet destruction or transfuse platelets. However, these choices are not always effective and may result in severe side effects (Portielje et al., 2001). For example, although splenectomy is effective in quite a few patients with thrombocytopenia, the risks of the operation itself and the various postoperative complications, such as severe sepsis, cannot be ignored (George, 2006). Platelet transfusion may cause immune responses (fever, allergy, and hemolysis) and non-immune responses (circulatory overload and bacterial contamination) (Li and Zheng, 2014). Thus, in contrast with mature therapies that attach importance to reducing platelet destruction and transfusing platelets, the stimulation of platelet formation is a newer approach (Siegal et al., 2013).

Although platelet development has been shown to be regulated by many growth factors and cytokines, thrombopoietin (TPO) was found to be a major physiological regulator of platelet production (Kaushansky and Drachman, 2002). TPO is a hormone produced by the liver and secreted into the peripheral circulation system. It binds to, and activates, TPO receptors (c-Mpl) and specifically promotes platelet production (Kaushansky, 2005). In recent years, many screening methods have been developed to identify significant molecules that can imitate the function of hematopoietic growth factors. The potential advantages of these molecule mimetics include their hypothetical lack of immunogenicity and low cost (Fukushima-Shintani et al., 2009). For TPO, several small-molecule compounds have been reported to imitate the role of TPO through TPO receptors (Kuter, 2007). The TPO receptor agonist (TPO-RA) acts as an earlier recombinant thrombopoietin and will increase the platelet count and decrease the need for platelet transfusions (Kuter, 2013). The current response rates to TPO-RA in children are comparable to those for intravenous immunoglobulin, and it has been well tolerated with minor side effects. TPO-RA directly targets thrombocytopenia and has few off-target effects. However, they are representative of the paradigm shift in ITP treatment: TPO-RA does not have immunosuppressive effects and therefore avoids the side effects of traditional immunosuppressive therapies. Overall, TPO-RA represents a new and effective alternative therapy for thrombocytopenia (Garzon and Mitchell, 2015).

Two TPO-RAs, eltrombopag and romiplostim, have been approved for the treatment of thrombocytopenia. Both are effective, but increased the risk of thrombosis (Nguyen et al., 2015). Currently, in the United States, romiplostim must be administered subcutaneously by health care professionals, and patients receiving eltrombopag must adhere to dietary restrictions (US FDA, 2008; US FDA, 2014). Avatrombopag (AKR 501, YM477, AS1670542, E5501) belongs to the second generation of new oral non-peptide TPO-RAs and imitates the function of TPO *in vitro* and *in vivo*. Avatrombopag increased platelet count in animals and human volunteers (Fukushima-Shintani et al., 2009). The US Food and Drug Administration (FDA) approved Doptelet^®^ (avatrombopag tablet) on May 21, 2018, for the treatment of other thrombocytopenia disorders, including ITP and CLD-induced thrombocytopenia (Neunert et al., 2011). There is currently no comprehensive analysis of the efficacy and safety of avatrombopag in patients with thrombocytopenia. In this study, a computer-based search was used to retrieve randomized, double-blind clinical trials involving patients with thrombocytopenia and the relevant data were extracted to permit an evidence-based medical meta-analysis of the efficacy and safety of avatrombopag in thrombocytopenia.

## Materials and Methods

### Search Strategy

According to the requirements of Preferred Reporting Items for Systematic Reviews and Meta-Analyses (PRISMA) statement (Liberati et al., 2009), and Cochrane Handbook for Interventional Systematic Reviews (Higgins and Green, 2011), databases including Medline, PubMed, Embase, the Cochrane Library, and ClinicalTrials.gov were searched. Search terms and MeSH were “thrombocytopenia,” “avatrombopag,” “E5501,” “AKR-501,” “YM477,” and “AS1670542.” We registered this review in the PROSPERO (registration number: CRD42018102888).

### Study Selection

The titles and abstracts of all searched records were independently screened by two authors (CL and XL) independently to determine potentially eligible studies. Subsequently, eligible studies were identified after screening the full text of the article. The following records were excluded: reviews, case reports, non-clinical studies, and irrelevant data. When the two authors’ opinions were contradictory, a third author was included in the discussion to aid resolution.

### Inclusion and Exclusion Criteria

According to the diagnostic criteria of WHO, trials including the administration of avatrombopag to patients with thrombocytopenia were included if they met the following criteria: 1) *Research design:* randomized, double-blind, controlled trials and all trials comparing the effectiveness and side effects of thrombocytopenia with avatrombopag, placebo, or no treatment for thrombocytopenia were eligible. 2) *Patients:* a) male or female patients older than 18 years without thrombosis and cardiovascular disease; b) mean baseline platelet count of patients less than 50 × 10^9^/L or platelet count between 20 × 10^9^/L and 70 × 10^9^/L. 3) *Interventions:* oral administration of avatrombopag alone. 4) *Outcome measures:* we pre-specified four outcomes, numbers of patients achieved platelet response (PR), mean platelet count change, adverse events (AEs), serious adverse serious adverse events (SAEs), and deaths: a) PR (platelet count ≥ 50 × 10^9^/L) is the primary outcome; b) PR (platelet count ≥ 100 × 10^9^/L), mean platelet count change, AEs (including abdominal pain, pyrexia, headache, nausea, fatigue, and peripheral edema), SAEs (including thrombosis, acute myocardial infarction, and hypotension), and deaths. Trials were excluded for the following reasons: 1) document type setting (reviews, meeting summaries, letters, etc.), 2) the information on the trial was missing or incomplete, and 3) the method of random sequence generation was not mentioned.

### Data Analysis

Trials were pooled by meta-analysis with RevMan5.3. RR and OR with 95% CI were used to assess dichotomous variables. Continuous variables were analyzed by using WMD with 95% CI. The *I* ^2^ statistic was used to assess heterogeneity. A fixed effect model was used when *I* ^2^ < 50%, which indicated heterogeneity. If *I* ^2^ > 50%, a random effects model was used after consideration of the potential sources of heterogeneity.

### Evidence Quality Assessment

Evidence quality assessment was performed separately for each outcome. The GRADE system defines the quality of evidence as “high,” “moderate,” “low,” or “very low.” If the opinion of the two authors differed, the contradiction was resolved through discussion.

## Results

### Study Selection and Characteristics

**Figure 1:**
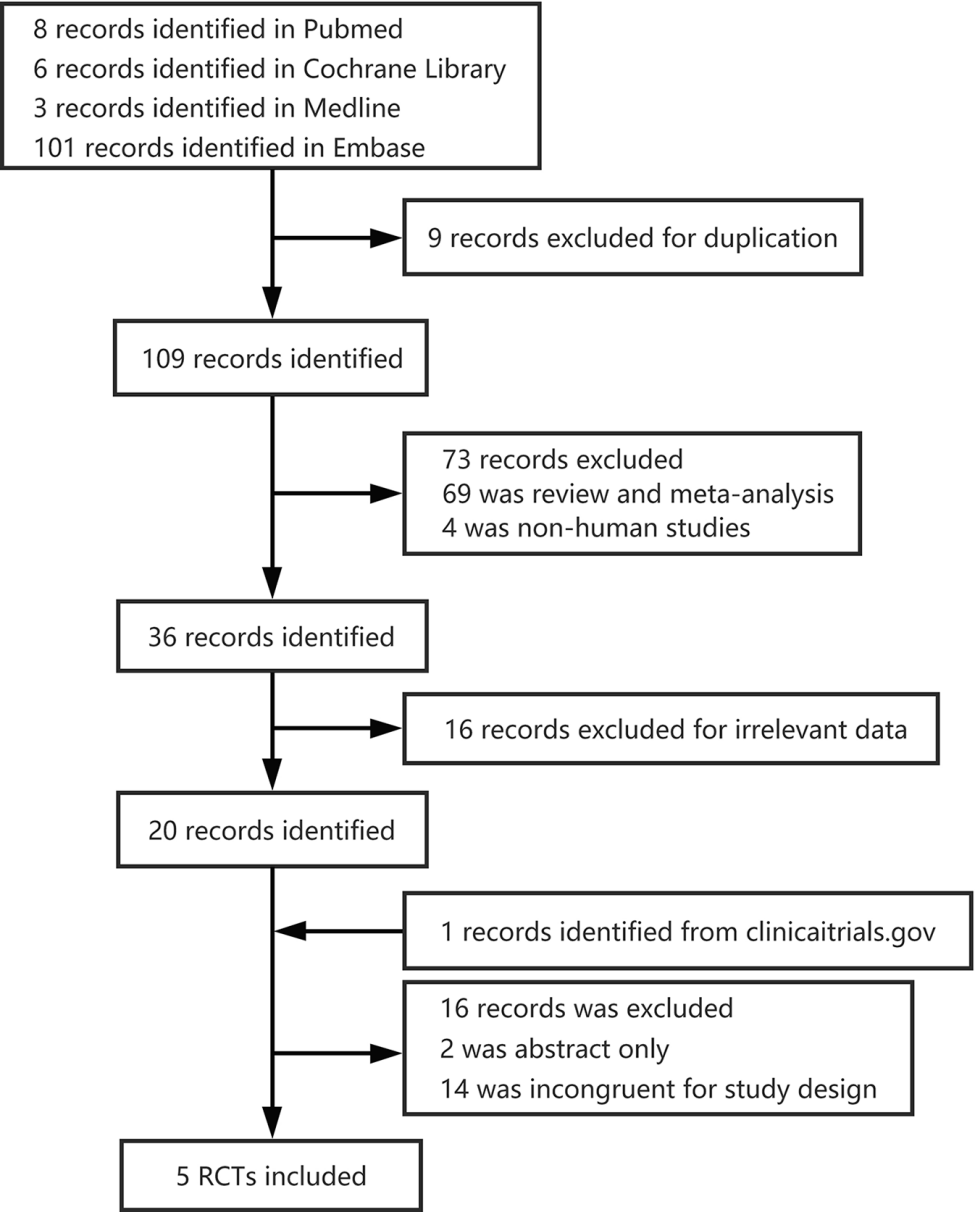
Flow chart of selected studies.

We screened four databases independently, and identified 8 PubMed publications, 6 Cochrane Library publications, 3 Medline publications, and 101 Embase publications for further study. From these, nine were excluded as duplicates, and 109 records remained. Twenty studies remained after the removal of reviews, meta-analyses, non-human studies, and irrelevant data. In total, five randomized controlled trials (RCTs) with 743 patients (Alireza, 2014; Bussel et al., 2014; Terrault et al., 2014; Jurczak et al., 2018; Terrault et al., 2018) were included in the meta-analysis (**Figure 1**). The studies analyzed 463 male patients and 280 female patients.

A summary of the baseline characteristics of the included RCTs is presented in **Table 1**. Four studies were fully published (Bussel et al., 2014; Terrault et al., 2014; Jurczak et al., 2018; Terrault et al., 2018); one study has been published in ClinicalTrials.gov only (Alireza, 2014). As one publication (Terrault et al., 2018) contained two RCTs, we divided it into two datasets (ADAPT1 and ADAPT2). We analyzed a total of six RCTs. Three of the included RCTs were phase II studies (Alireza, 2014; Bussel et al., 2014; Terrault et al., 2014) and the others were phase III studies (Jurczak et al., 2018; Terrault et al., 2018). Patients with thrombocytopenia and CLD were included in four of the RCTs (Alireza, 2014; Terrault et al., 2014; Terrault et al., 2018); the other two RCTs included patients with thrombocytopenia and/or ITP (Bussel et al., 2014; Jurczak et al., 2018). The baseline platelet count ranged from 36.15 to 40.9 × 10^9^/L, and the patients were, on average, between 39.6 and 58.28 years of age. Three trials were conducted in multiple countries (Terrault et al., 2014; Jurczak et al., 2018; Terrault et al., 2018); the others were conducted in the United States. For all included RCTs, avatrombopag and placebo were orally administered once daily as a monotherapy. For analysis, we combined the different dosing regimens because different concentrations of avatrombopag were administered to patients in the included RCTs.

**Table 1:** Characteristics of randomized controlled trials.

Study, year	Participants	Gender	Interventions	Age (years)	Baseline platelet count (10^9^/L)	Phase	Location	Study sponsor
Jurczak et al., 2018	*N* = 49	M: 36.7% F: 63.3%	Avatrombopag 6 months Placebo 6 months	46.40 ± 4.20 41.20 ± 4.70	NR	3	Australia, Belgium, Bulgaria, Czechia, Netherlands, New Zealand, Poland, Singapore, Slovakia, South Africa, Ukraine.	Eisai Inc.
NCT01355289 2018	*N* = 65	M: 73.8% F: 26.2%	Avatrombopag 21 days Placebo 21 days	54.65 ± 7.45 50.20 ± 7.96	NR	2	United States.	Eisai Inc.
Terrault et al., 2018(ADAPT-1)	*N* = 231	M: 68.4% F: 31.6%	Avatrombopag 5 days Placebo 5 days	56.35 ± 9.52 56.22 ± 1.05	36.15 ± 8.58 36.80 ± 8.96	3	United States, Argentina, Australia, Austria, Belgium, Brazil, Canada, Chile, China, France, Germany, Hungary, Italy, Korea, Poland, Portugal, Spain, Thailand, United Kingdom.	Eisai Inc.
Terrault et al., 2018(ADAPT-2)	*N* = 204	M: 62.3% F: 37.7%	Avatrombopag 5 days Placebo 5 days	58.28 ± 2.84 58.13 ± 1.25	37.98 ± 7.14 38.21 ± 7.74	3	United States, Argentina, Australia, Belgium, Brazil, Canada, China, Czechia, France, Germany, Israel, Italy, Japan, Mexico, Romania, Russian Federation, Spain.	Eisai Inc.
Bussel et al., 2014	*N* = 64	M: 37.5% F: 62.5%	Avatrombopag 28 days Placebo 28 days	53.41 ± 7.50 39.60 ± 0.63	NR	2	United States	Eisai Inc.
Terrault et al., 2014	*N* = 130	M: 67.7% F: 32.3%	Avatrombopag 7 days Placebo 7 days	54.87 ± 6.56 54.99 ± 6.62	40.90 ± 9.48 38.00 ± 8.52	2	United States	Eisai Inc.

M, male; F, female; NR, not report; NCT01355289: This study has not been published.

Inclusion criteria: patients in the study must have a platelet count less than 50 × 10^9^/L or platelet count greater than or equal to 20 × 10^9^/L to 70 × 10^9^/L.

### Risk of Bias

**Figure 2:**
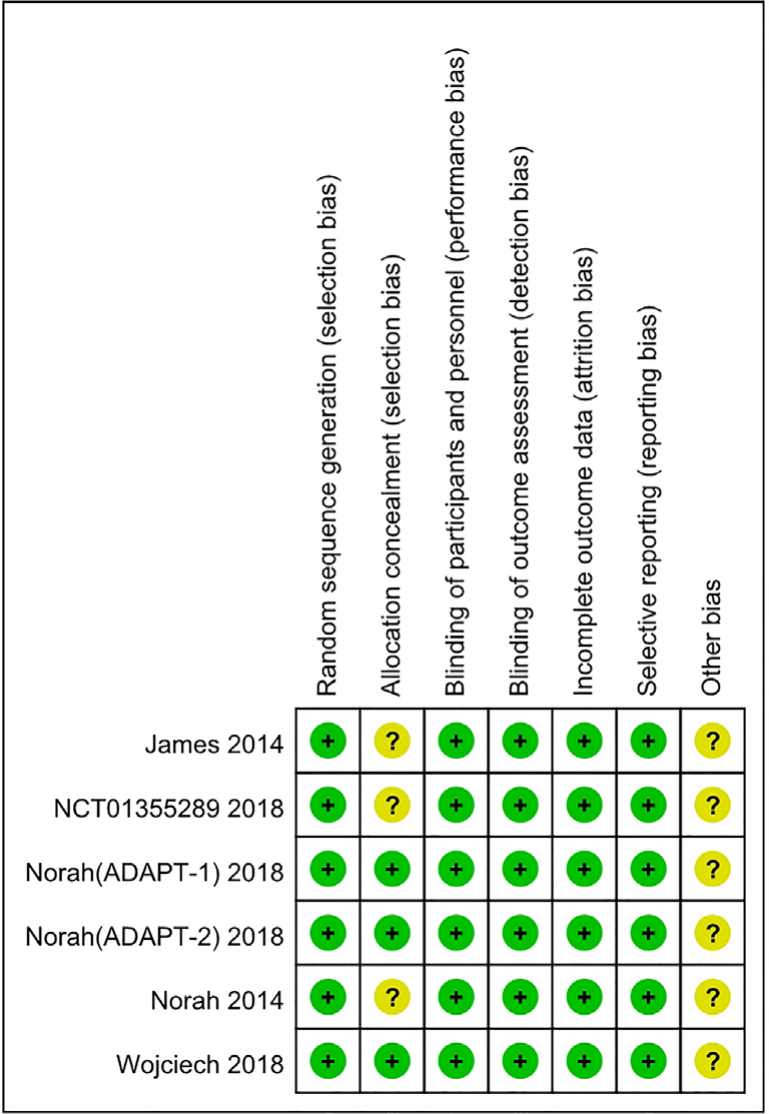
Risk of bias summary.

**Figure 3:**
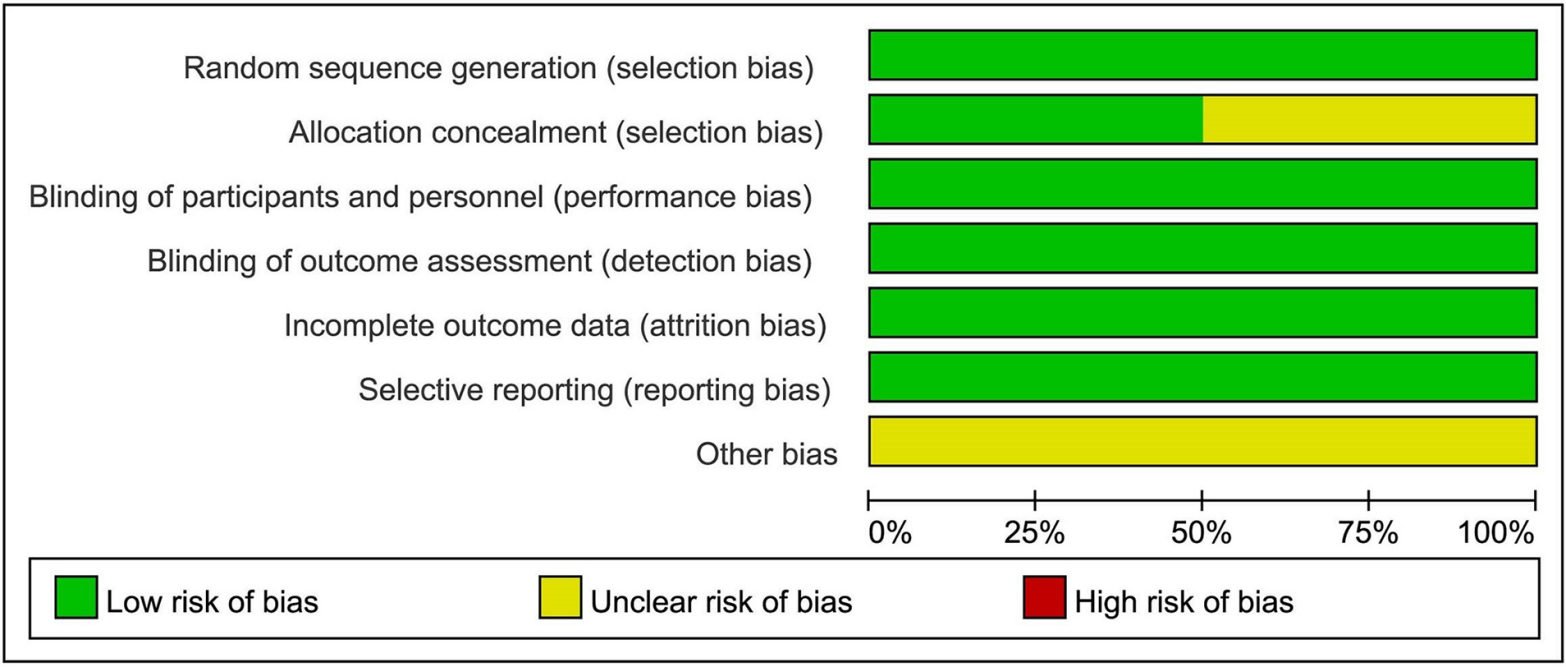
Risk of bias graph.

Although the RCTs were small, they were judged to be of high quality (**Figures 2** and **3**). The generation of random sequences was described in detail for all RCTs, and the method of allocation concealment was described in three RCTs. The blinding was at a low risk of bias. All trials were sponsored by Eisai Inc. (New Jersey, United States). We strongly suspected the credibility. However, after comparing the outcomes pre-specified in the protocols and those reported, we judged that selective reporting was at low risk of bias.

### Data Extraction and Risk of Bias Assessment

Two authors (CL and XL) extracted the data independently to complete the extraction table. The variables included in the extraction table were as follows: author, date, total number of participants, sex, age, disease, interventions, mean platelet count, the number of participants who achieved PR ≥ 50 × 10^9^/L or 100 × 10^9^/L, AEs, SAEs, and deaths. The risk of bias tool recommended by Cochrane was used to assess the selection bias (random sequence generation and allocation concealment), performance bias (blinding of participants and personnel), detection bias (blinding of outcome assessment), attrition bias (incomplete outcome data), reporting bias (selective reporting), and other bias of the included trials. The quality of risk assessment was judged as “low risk,” “unclear risk,” or “high risk.”

### PR

**Figure 4:**
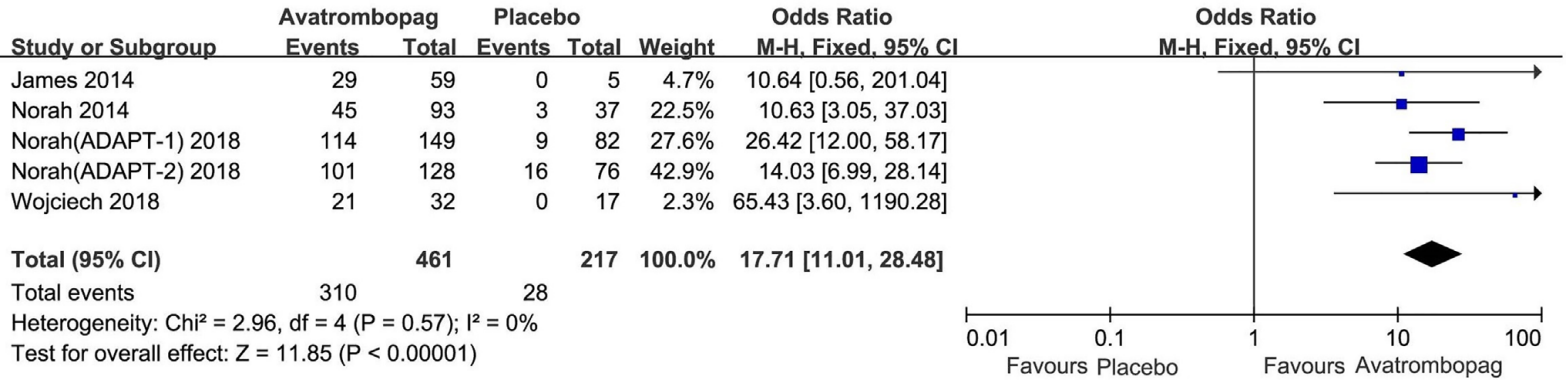
Forest plot: The number of patients who achieved PR ≥ 50 × 10^9^/L for avatrombopag versus placebo from the meta-analysis.

**Figure 5:**
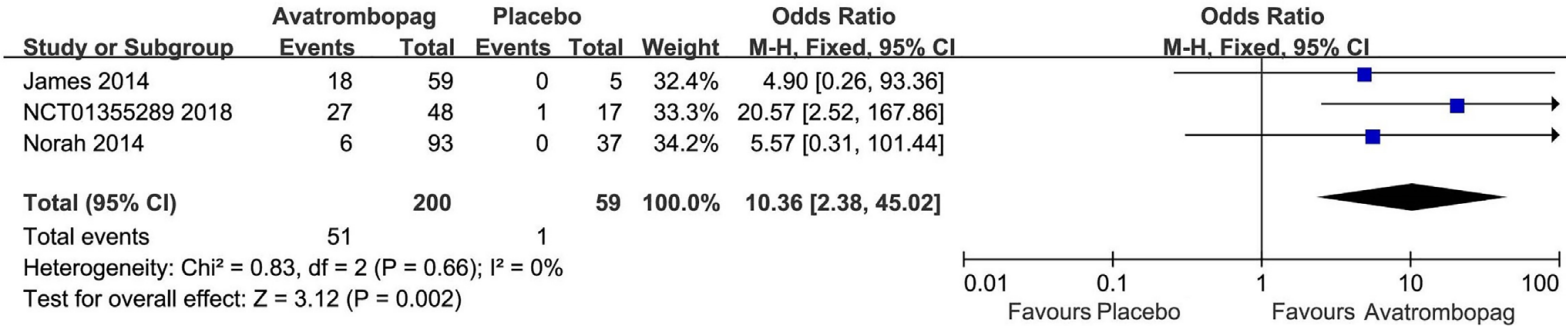
Forest plot: The number of patients who achieved PR ≥ 100 × 10^9^/L with avatrombopag treatment versus placebo from the meta-analysis.

The effects of avatrombopag versus placebo on PR are shown in **Figures 4** and **5**, respectively. Five trials used the outcome of PR ≥ 50 × 10^9^/L. More patients who received avatrombopag achieved the aim of PR ≥ 50 × 10^9^/L (OR: 17.71, 95% CI [11.01 to 28.48], *p* < 0.00001) than those who received placebo (**Figure 4**). The level of heterogeneity was low for these five trials, with an *I* ^2^ of 0 and the 95% CI was narrow. Only three trials used the outcome of PR ≥ 100 × 10^9^/L. More patients who received avatrombopag achieved the aim of PR ≥ 100 × 10^9^/L (OR: 10.36, 95% CI [2.38 to 45.02], *p* = 0.002) than those who received placebo (**Figure 5**). The level of heterogeneity was low for these three trials, with an *I* ^2^ of 0, although the 95% CI was wide.

### Change in Platelet Count From Baseline

**Figure 6:**
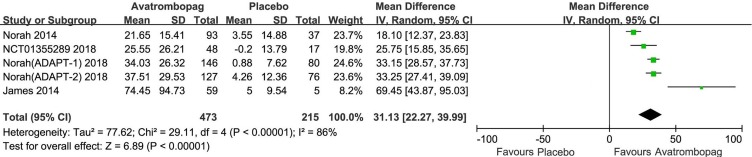
Forest plot: Effects of avatrombopag and placebo on platelet count change from the baseline from the meta-analysis.

**Figure 7:**
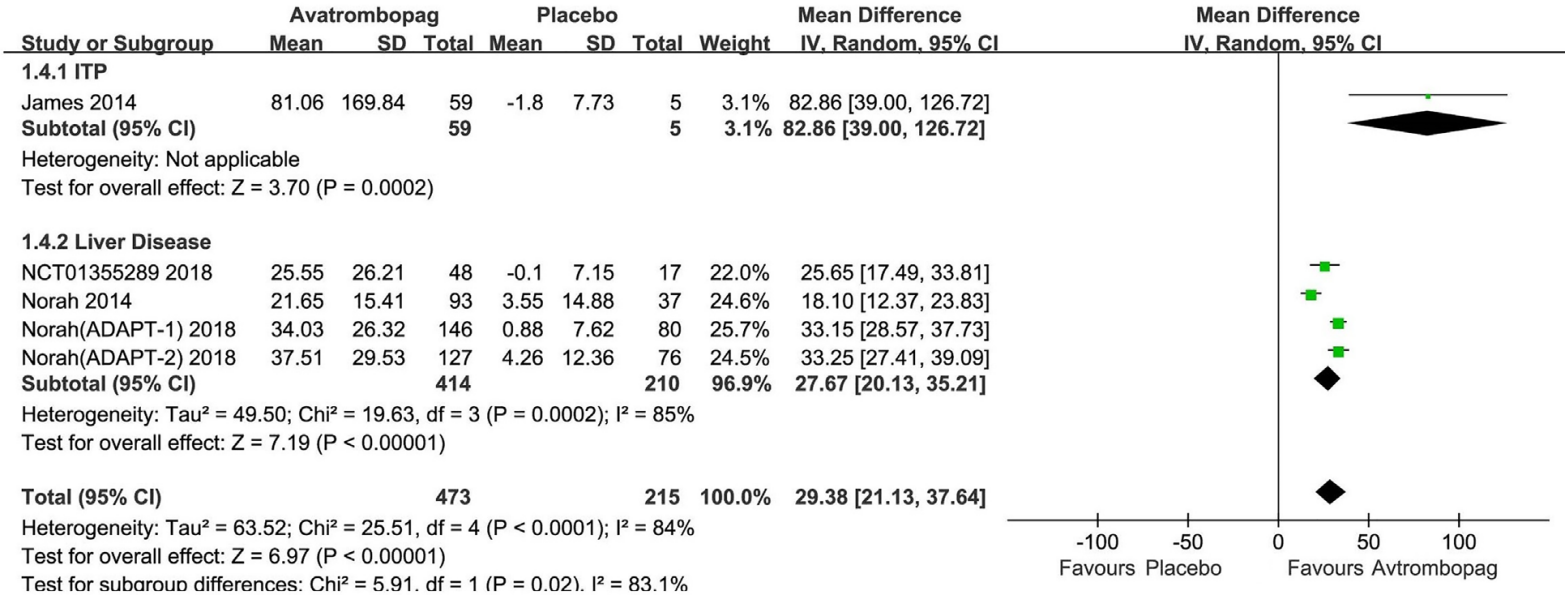
Forest plot: Effects of avatrombopag and placebo on platelet count in the presence of different comorbidities.

Avatrombopag significantly the increased platelet count (WMD: 31.13%, 95% CI [22.27 to 39.99], *p* < 0.00001) unlike the placebo (**Figure 6**). The level of heterogeneity was very high and was not reduced, even when we analyzed the subgroups with different diseases (**Figure 7**). Two studies (Bussel et al., 2014; Terrault et al., 2014) were removed given their larger or smaller effect size than other RCTs; subsequently, the level of heterogeneity was reduced and the 95% CI narrowed significantly (WMD: 31.96%, 95% CI [28.66 to 35.25], *p* < 0.00001; *I* ^2^ = 27%).

### SAEs and Deaths

**Figure 8:**
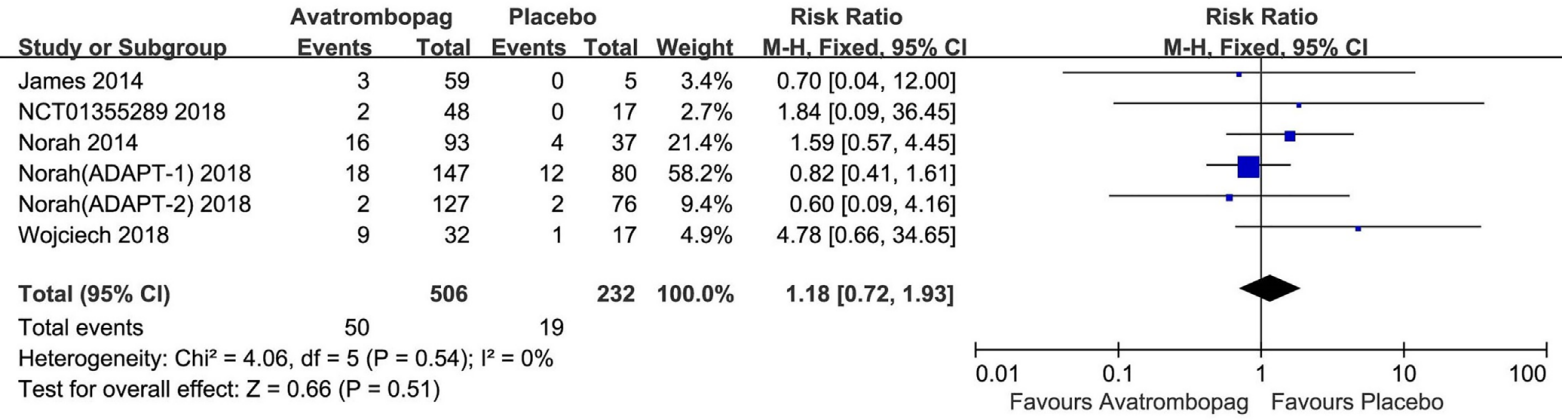
Forest plot: The incidence of serious adverse events (SAEs) after avatrombopag and placebo treatment from the meta-analysis.

**Figure 9:**
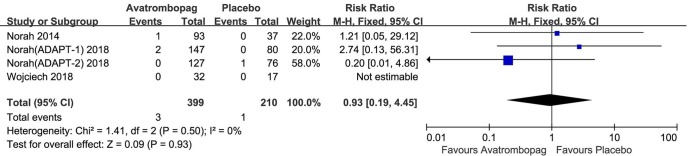
Forest plot: The incidence of deaths after avatrombopag and placebo treatment from the meta-analysis.

The SAEs and deaths of patients administered avatrombopag and placebo are shown in **Figures 8** and **9**, respectively. The pooled estimate showed no significant difference in SAEs for avatrombopag versus placebo (RR: 1.18, 95% CI [0.72 to 1.93], *p* > 0.05) (**Figure 8**). Deaths were reported in three RCTs during the study period. No deaths occurring during the study was reported by one RCT. The meta-analysis was used for the estimation of a non-significant improvement in the probability of deaths in patients administered avatrombopag versus placebo (RR: 0.93, 95% CI [0.19 to 4.45], *p* > 0.05) (**Figure 9**).

### Other AEs

**Figure 10:**
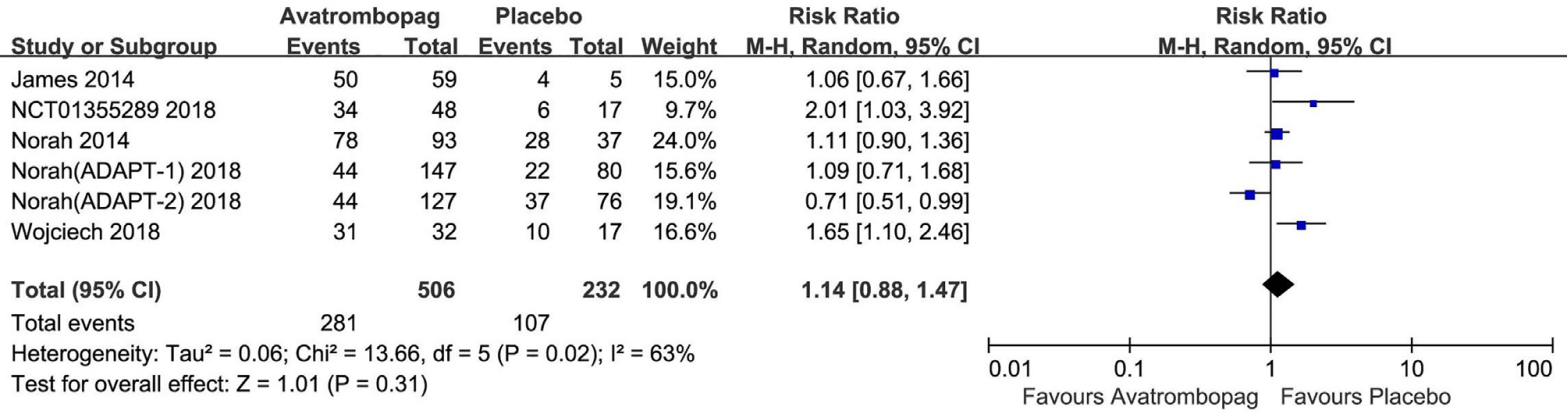
Forest plot: The incidence of other AEs after avatrombopag and placebo treatment from the meta-analysis.

The result showed no significant difference in AEs in patients administered avatrombopag versus placebo (RR: 1.14, 95% CI [0.88 to 1.47], *p* > 0.05, *I* ^2^ = 63%) (**Figure 10**). Owing to the high heterogeneity, we removed one RCT [Terrault et al., 2018 (ADAPT2) ] because of the different effect to other trials; subsequently, heterogeneity was reduced and the 95% CI changed significantly (RR: 1.25, 95% CI [1.05 to 1.49], *p* = 0.01; *I* ^2^ = 27%). Thus, the results showed a slight difference for AEs after the administration of avatrombopag compared to placebo.

### GRADE

A GRADE evidence profile was created to evaluate the quality of evidence for the key outcomes (**Table 2**). For most outcomes, we downgraded the imprecision by one because the 95% CIs were wide and there were less than 300 samples in total. The GRADE results showed that the evidence was “low” for most outcomes and “moderate” for a few outcomes.

**Table 2:** GRADE assessment of the quality of the included studies.

Quality assessment							No. of patients		Effect		Quality	Importance
No. of studies	Design	Risk of bias	Inconsistency	Indirectness	Imprecision	Other considerations	Avatrombopag	Placebo	Relative (95% CI)	Absolute		
**≥50 × 10** **^9^** **/L**												
5	Randomized trials	No serious risk of bias	No serious inconsistency	No serious indirectness	Serious^1^	None	310/461 (67.2%)	28/217 (12.9%)	RR 5.61 (4.81 to 6.27)	595 more per 1,000 (from 492 more to 680 more)	MODERATE	CRITICAL
								8.1%		373 more per 1,000 (from 309 more to 427 more)		
**≥100 × 10** **^9^** **/L**												
3	Randomized trials	No serious risk of bias	No serious inconsistency	No serious indirectness	Very serious^1,2^	None	51/200 (25.5%)	1/59 (1.7%)	RR 8.94 (2.33 to 25.75)	135 more per 1,000 (from 23 more to 419 more)	LOW	IMPORTANT
								0%		-		
**Change in platelet count from baseline**												
5	Randomized trials	No serious risk of bias	Serious^3^	No serious indirectness	Serious^1^	None	473	215	-	MD 31.13 higher (22.27 to 39.99 higher)	LOW	NOT IMPORTANT
**Change from baseline in platelet count** (**different disease**)												
4	Randomized trials	No serious risk of bias	Serious^3^	No serious indirectness	Serious^1^	None	380	178	-	MD 32.2 higher (25.82 to 38.58 higher)	LOW	NOT IMPORTANT
**Change from baseline in platelet count—ITP** (**better indicated by lower values**)												
1	Randomized trials	No serious risk of bias	Serious^4^	No serious indirectness	Serious^1,2^	None	59	5	-	MD 82.86 higher (39 to 126.72 higher)	LOW	NOT IMPORTANT
**Change from baseline in platelet count—liver disease** (**better indicated by lower values**)												
3	Randomized trials	No serious risk of bias	Serious^3^	No serious indirectness	Serious^1^	None	321	173	-	MD 31.68 higher (27.69 to 35.68 higher)	MODERATE	NOT IMPORTANT
**SAEs**												
6	Randomized trials	No serious risk of bias	No serious inconsistency	No serious indirectness	Serious^1^	None	50/506 (9.9%)	19/232 (8.2%)	RR 1.19 (0.71 to 1.93)	16 more per 1,000 (from 24 fewer to 76 more)	LOW	CRITICAL
								4.3%		8 more per 1,000 (from 12 fewer to 40 more)		
**Other AEs**												
6	Randomized trials	No serious risk of bias	No serious inconsistency	No serious indirectness	Serious^1^	None	281/506 (55.5%)	107/232 (46.1%)	RR 1.29 (0.86 to 1.66)	134 more per 1,000 (from 65 fewer to 304 more)	LOW	CRITICAL
								53.8%		156 more per 1,000 (from 75 fewer to 355 more)		
**Death events**												
4	Randomized trials	No serious risk of bias	Serious^3^	No serious indirectness	Serious^1^	None	3/399 (0.75%)	1/210 (0.48%)	RR 0.93 (0.19 to 4.4)	0 fewer per 1,000 (from 4 fewer to 16 more)	MODERATE	CRITICAL
								0%		-		

^1^Large variation in confidence interval.

^2^Very small sample size.

^3^The p value for heterogeneity is less than 0.05, and I^2^ > 60%.

^4^Only one study.

## Discussion

Avatrombopag is a promising therapeutic agent for thrombocytopenia, and thrombocytopenia represents an important clinical challenge. It is the second generation of the orally bioavailable and small-molecule TPO-RA created for the treatment of thrombocytopenia by Dova Pharmaceuticals (US FDA, 2018). In summary, the available clinical data support the efficacy of avatrombopag. As with all new drugs, further monitoring and evaluation are needed to determine the effects of avatrombopag treatment in more patients for longer periods of time. This study has included available clinical experimental data for the treatment of avatrombopag in thrombocytopenia and used systematic reviews and meta-analyses to evaluate its efficacy and safety.

Our results show that avatrombopag is effective for the treatment of thrombocytopenia. Avatrombopag significantly improves overall PR and platelet count. To increase peripheral blood platelet count to normal levels is the main goal of treatment for thrombocytopenia (Lo and Deane, 2014). Platelet response is the primary outcome measure, which is defined as the number of patients who achieve a platelet count of ≥50 × 10^9^/L (Newland et al., 2016). We also included the outcome measure of the patients who achieved a platelet count ≥100 × 10^9^/L. The meta-analysis results showed that more patients treated with avatrombopag achieved platelet than those treated with placebo. The platelet counts of patients treated with avatrombopag showed a greater change from baseline than in patients treated with the placebo. However, owing to the high heterogeneity, we performed a subgroup analysis and removed two trials; consequently, the heterogeneity decreased to 27%. We believed that the high heterogeneity occurred because the therapeutic effect of avatrombopag on thrombocytopenia caused by different types of diseases was varied. ITP is an autoimmune disorder characterized by persistent thrombocytopenia due to the binding of autoantibody to platelets (British Committee for Standards in Haematology General Haematology Task Force, 2003). The main mechanism of thrombocytopenia in liver diseases is the retention of platelets in the spleen and the decrease of TPO in the liver Samuel et al., (2016). Therefore, for thrombocytopenia arising from different disease backgrounds, the effects should be analyzed separately. However, we cannot obtain more reliable results because the trials included in this study were small.

The risk of adverse events in patients treated with avatrombopag was the same as in patients treated with placebo. However, after removal of trial with differing results, the risk of adverse events in patients treated with avatrombopag was higher than in patients treated with placebo. This trial demonstrated an improvement in adverse events and an opposite result to other trials, so we removed it. After this trial was removed, the meta-analysis results indicated that patients treated with avatrombopag may experience adverse events. Most common adverse events include abdominal pain, pyrexia, headache, nausea, fatigue, peripheral edema, etc. (Shirley, 2018). We found no statistically significant difference in the risk of developing any severe adverse events and death events in avatrombopag and placebo. The criteria for severe adverse effects and adverse effects were shown in these included trials; some severe adverse events include thrombosis, acute myocardial infarction, hypotension, etc. (Alireza, 2014; Bussel et al., 2014; Terrault et al., 2014; Jurczak et al., 2018; Terrault et al., 2018). Thrombotic events have been reported with eltrombopag and romiplostim. Both arterial and venous thromboses have been described (Kuter et al., 2008; Kuter, 2013; Saleh et al., 2013). The thrombotic events in these studies were attributed to the high (>200 × 10^9^/L) and sustained increase in platelet count (Afdhal et al., 2012). However, thrombosis was mentioned in only a few experiments in this meta-analysis, and the effect of avatrombopag on thrombosis remains unclear. There was a high level of heterogeneity and a lack of long-term data in included trials, which may affect our analysis. Therefore, large-scale, rigorously designed, multi-center randomized clinical trials are needed to verify the efficacy and safety of avatrombopag. In summary, the current studies have demonstrated that avatrombopag has a beneficial effect in patients with thrombocytopenia, and this meta-analysis shows that the treatment of avatrombopag results in significantly increased platelet count in patients with thrombocytopenia. Avatrombopag represents a promising therapeutic option for patients with thrombocytopenia. The early results for avatrombopag are encouraging, and we are looking forward to longer-term data to comprehensively assess the efficacy and risk of avatrombopag.

## Conclusion

This meta-analysis showed that avatrombopag is an effective treatment for thrombocytopenia, but there are sufficient evidences to demonstrate that its use may cause adverse events.

## Data Availability

Publicly available datasets were analyzed in this study. This data can be found here: https://www.ncbi.nlm.nih.gov/pubmed

## Author Contributions

CL, XL, WZ, and JW conceived and designed the review; CL, XL, FH, JY, AW, LW, DQ, WZ, and JW reviewed the literature; CL, XL, WZ, and JW wrote the manuscript.

## Funding

This work was supported by grants from the National Natural Science Foundation of China (Nos. 81774013 and 81804221), the Science and Technology Planning Project of Sichuan Province, China (Grant Nos. 2018JY0237 and 2019YJ0484), Educational Commission of Sichuan Province, China (Grant Nos. 18TD0051 and 18ZA0525), Administration of traditional Chinese medicine in Sichuan Province, China (Grant Nos. 2018JC013 and 2018JC038), Science and Technology Program of Luzhou, China (Grant Nos. 2017-S-39(3/5), 2018LZXNYD-ZK31, and 2016LZXNYD-T03), and the Key Development Program of Southwest Medical University (Grant Nos. 2017-ZRQN-081 and 2017-ZRZD-017).

## Conflict of Interest Statement

The authors declare that the research was conducted in the absence of any commercial or financial relationships that could be construed as a potential conflict of interest.
